# FAM3C activates HSF1 to suppress hepatic gluconeogenesis and attenuate hyperglycemia of type 1 diabetic mice

**DOI:** 10.18632/oncotarget.22524

**Published:** 2017-11-20

**Authors:** Zhenzhen Chen, Junpei Wang, Weili Yang, Ji Chen, Yuhong Meng, Biaoqi Feng, Yujing Chi, Bin Geng, Yong Zhou, Qinghua Cui, Jichun Yang

**Affiliations:** ^1^ Hypertension Center, Fuwai Hospital, Chinese Academy of Medical Sciences and Peking Union Medical College, State Key Laboratory of Cardiovascular Disease, National Center for Cardiovascular Diseases, Beijing 100037, China; ^2^ Department of Physiology and Pathophysiology, School of Basic Medical Sciences, Key Laboratory of Molecular Cardiovascular Science of The Ministry of Education Center for Non-Coding RNA Medicine, Peking University Health Science Center, Beijing 100191, China; ^3^ Institute of Clinical Molecular Biology & Central Laboratory, Peking University People's Hospital, Beijing 100044, China; ^4^ Beijing Institute of Heart, Lung and Blood Vessel Diseases, Beijing Anzhen Hospital, Capital Medical University, Beijing 100029, China; ^5^ Department of Biomedical Informatics, School of Basic Medical Sciences, Key Laboratory of Molecular Cardiovascular Science of The Ministry of Education, Center for Non-Coding RNA Medicine, Peking University Health Science Center, Beijing 100191, China

**Keywords:** FAM3C, hepatokine, HSF1, type 1 diabetes, gluconeogenesis

## Abstract

FAM3C, a member of FAM3 gene family, has been shown to improve insulin resistance and hyperglycemia in obese mice. This study further determined whether FAM3C functions as a hepatokine to suppress hepatic gluconeogenesis of type 1 diabetic mice. In STZ-induced type 1 diabetic mouse liver, the FAM3C-HSF1-CaM signaling axis was repressed. Hepatic FAM3C overexpression activated HSF1-CaM-Akt pathway to repress gluconeogenic gene expression and ameliorate hyperglycemia of type 1 diabetic mice. Moreover, hepatic HSF1 overexpression also activated CaM-Akt pathway to repress gluconeogenic gene expression and improve hyperglycemia of type 1 diabetic mice. Hepatic FAM3C and HSF1 overexpression had little effect on serum insulin levels in type 1 diabetic mice. In cultured hepatocytes, conditioned medium of Ad-FAM3C-infected cells induced Akt phosphorylation. Moreover, Akt activation and gluconeogenesis repression induced by FAM3C overexpression were reversed by the treatment with anti-FAM3C antibodies. Treatment with recombinant FAM3C protein induced Akt activation in a HSF1- and CaM-dependent manner in cultured hepatocytes. Furthermore, recombinant FAM3C protein repressed gluconeogenic gene expression and gluconeogenesis by inactivating FOXO1 in a HSF1-dependent manner in cultured hepatocytes. In conclusion, FAM3C is a new hepatokine that suppresses hepatic gluconeogenic gene expression and gluconeogenesis independent of insulin by activating HSF1-CaM-Akt pathway.

## INTRODUCTION

Diabetes has become a severe worldwide epidemic affecting more than 400 million people [1]. Excessive hepatic gluconeogenesis due to insulin resistance or insulin deficiency plays crucial roles in the development of diabetes [2]. Type 1 diabetes is mainly due to auto-immune-mediated pancreatic beta cell destruction, resulting in the deficiency of circulating insulin [3]. FOXO1 is the key transcriptor regulating gluconeogenesis, and it is phosphorylated and inactivated by insulin-mediated Akt activation in physiological condition [4]. Overactivation of hepatic FOXO1 due to insulin deficiency or insulin resistance will trigger excessive gluconeogenesis and hyperglycemia. Insulin and insulin analogues are the main therapeutical agents for type 1 diabetes [5]. However, it had been revealed that drugs that suppress hepatic gluconeogenesis also help reduce blood glucose levels in type 1 diabetic patients. For example, adjunctive metformin therapy with insulin reduces HbA1c levels and daily insulin dosage [6, 7]. Identifying new genes or pathways that activate Akt to repress hepatic FOXO1 activity independent of insulin will shed light on the treatment of both type 1 and type 2 diabetes.

Family with sequence similarity 3 (FAM3) gene family consists of four members designated as FAM3A, FAM3B, FAM3C and FAM3D, respectively [8]. In the past decade, FAM3B, also called pancreatic derived factor (PANDER), had been revealed to be a negative regulator of pancreatic beta cell function and liver insulin signaling [9–14]. FAM3A is a novel mitochondrial protein that plays important roles in suppressing hepatic gluconeogenesis and lipogenesis by activating the ATP-P2 receptor-Akt pathway [15–17]. FAM3A also protect against liver ischemia/reperfusion injury [18]. More recently, we had demonstrated that FAM3C expression is reduced in the livers of obese mice. Hepatic FAM3C overexpression improves insulin resistance, hyperglycemia and steatosis of obese mice [19]. Mechanistically, FAM3C activates transcriptor heat shock factor 1 (HSF1) to induce CALM1 gene expression and increase calmodulin (CaM) protein level. An increase in CaM protein level finally suppresses glucose production by inducing Akt activation in hepatocytes [19]. Clearly, FAM3C represents a new target for treating type 2 diabetes. However, several issues still remain unaddressed in our previous study. One is that although FAM3C activates HSF1-CaM pathway to activate Akt independent of insulin in cultured hepatocytes, whether it can suppress hepatic gluconeogenesis to improve hyperglycemia of type 1 diabetic mice remains unknown. Generally, two FAM3C protein isoforms exist in hepatocytes [19], one is the full length form (FL, 26kD) and the other is the secretory form (SF, 22kD) [19, 20]. So, another important issue is that whether secretion is necessary for FAM3C's effects of activating Akt and suppressing gluconeogenesis in hepatocytes, particularly in the condition of FAM3C overexpression or upregulation. Addressing these issues is necessary for the establishment that FAM3C functions as a new hepatokine to suppress hepatic gluconeogenesis independent of insulin.

In the current study, we demonstrated that hepatic FAM3C or HSF1 overexpression repressed gluconeogenic gene expression and attenuated hyperglycemia of type 1 diabetic mice. FAM3C secretion is necessary for its effects of activating Akt and repressing gluconeogenesis in hepatocytes. Treatment with recombinant FAM3C protein (rFAM3C) repressed gluconeogenic gene expression and gluconeogenesis by activating Akt to repress FOXO1 activity in a HSF1-CaM-dependent manner in hepatocytes. Overall, these findings revealed that FAM3C is a new hepatokine that suppresses hepatic gluconeogenesis independent of insulin by activating HSF1-CaM-Akt pathway.

## RESULTS

### FAM3C-HSF1-CaM pathway was repressed in the livers of type 1 diabetic mice

**Figure 1:**
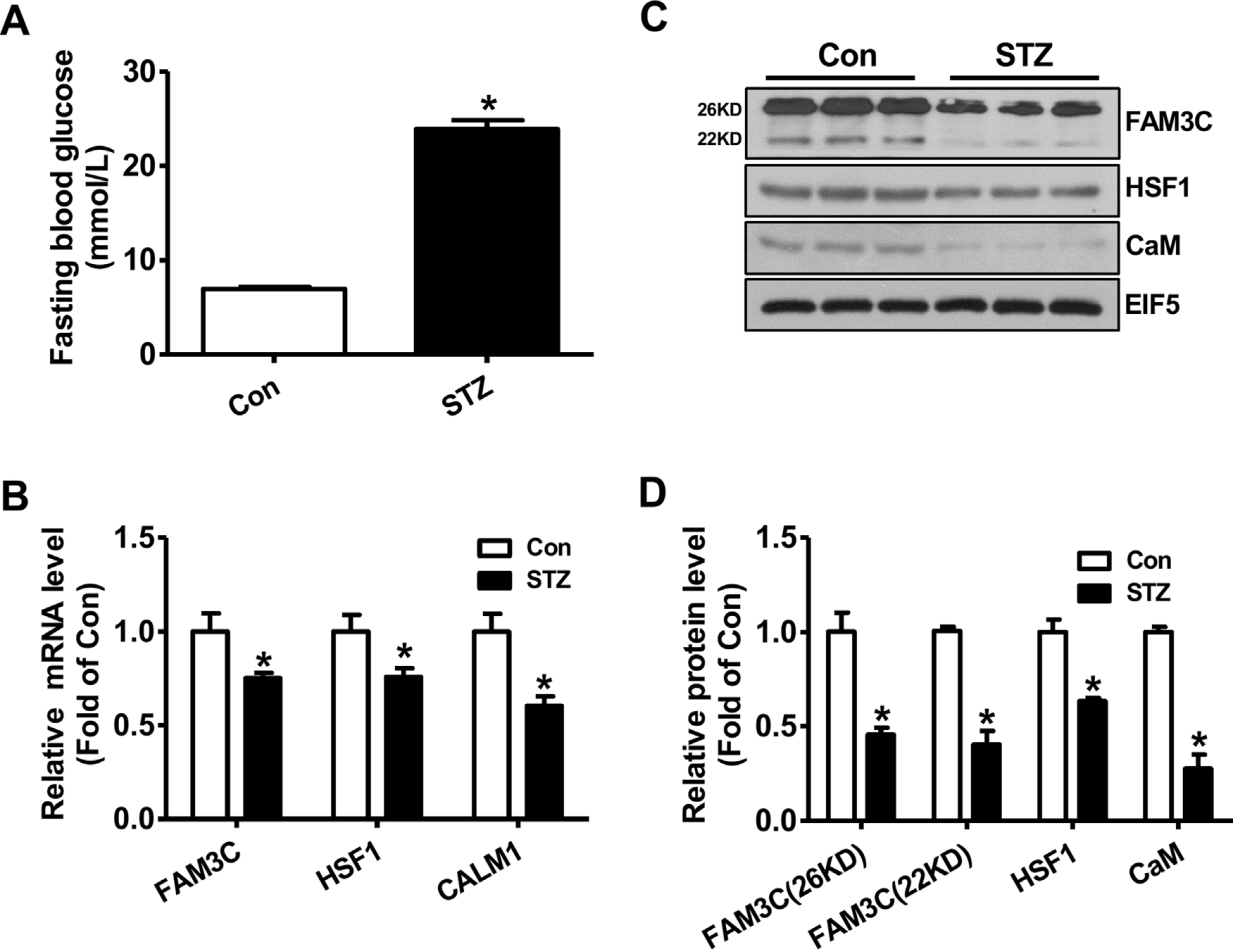
FAM3C-HSF1-CaM pathway was repressed in type 1 diabetic mouse livers **(A)** Fasting blood glucose levels of mice were markedly elevated at 1 month post STZ injection. **(B)** The mRNA levels of FAM3C, HSF1 and CALM1 were reduced in diabetic mouse livers. **(C-D)** The protein levels of FAM3C, HSF1 and CaM were reduced in diabetic mouse livers. The representative gel images were shown in panel C, and quantitative data in panel D. Con, control mice treated with saline; STZ, mice treated with STZ. N=6-8, ^*^P<0.05 versus control mice.

To determine whether FAM3C-HSF1-CaM pathway is involved in increased hepatic glucose production under type 1 diabetic condition, their expression levels in STZ-treated diabetic mouse livers were analyzed. One month later, the fasting blood glucose levels of STZ-treated mice were markedly elevated when compared with control mice (Figure 1A). In type 1 diabetic mouse livers, the mRNA levels of FAM3C, HSF1 and CALM1 were reduced when compared with control mouse livers (Figure 1B). In support, the protein levels of both FAM3C isoforms, HSF1 and CaM were also reduced in type 1 diabetic mouse livers when compared with normal mouse livers (Figure 1C-1D).

### Hepatic overexpression of FAM3C or HSF1 attenuated fasting hyperglycemia of type 1 diabetic mice

**Figure 2:**
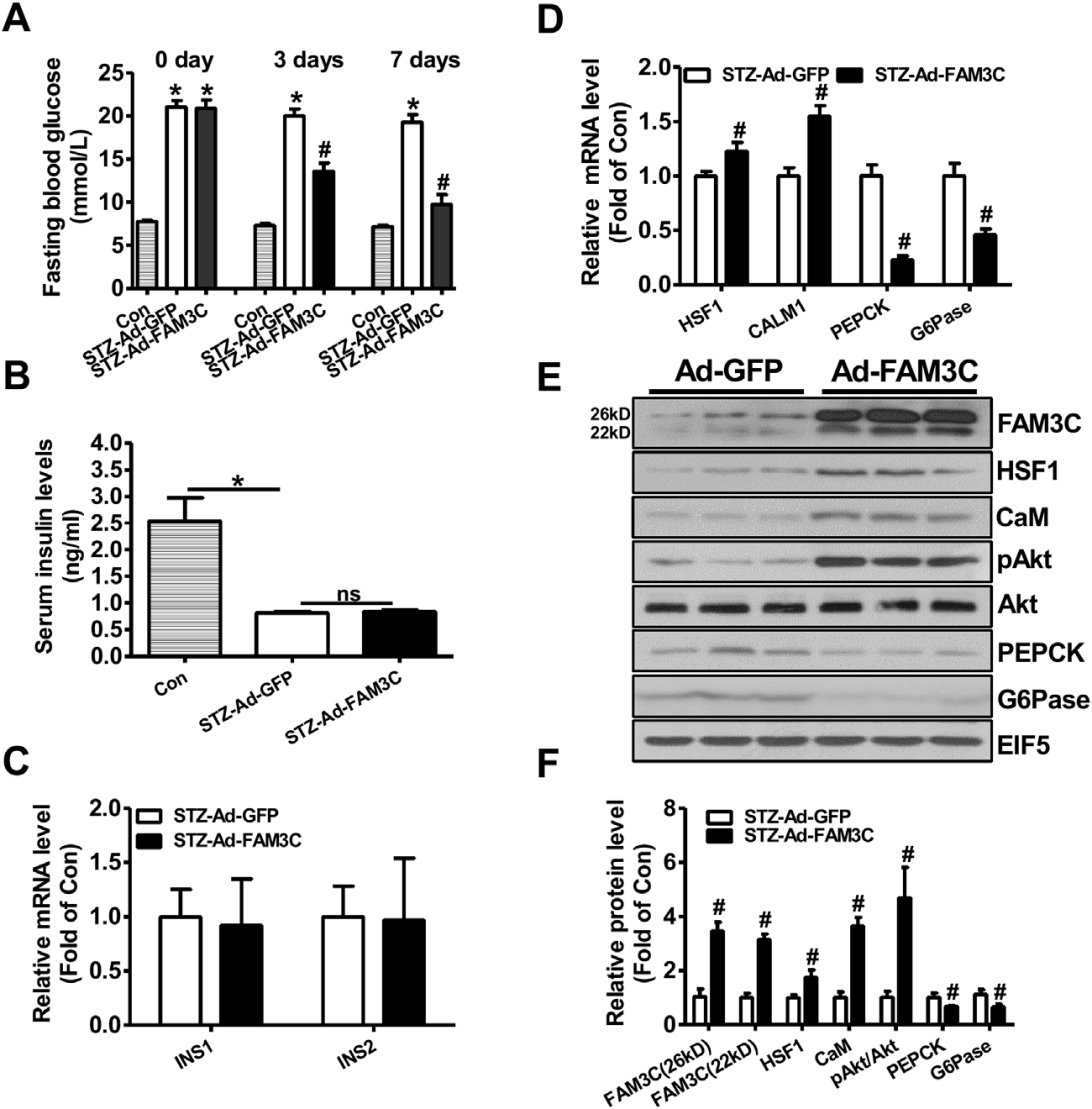
Adenoviral overexpression of FAM3C in type 1 diabetic mouse livers attenuated hyperglycemia Mice were transduced with Ad-FAM3C or Ad-GFP as detailed in the experimental procedure. **(A)** Fasting blood glucose levels of type 1 diabetic mice before and after Ad-FAM3C injection. **(B)** Serum insulin levels after Ad-FAM3C injection. **(C)** Insulin mRNA expression levels in pancreas of mice after Ad-FAM3C injection. **(D)** FAM3C overexpression on the mRNA levels of metabolic genes in diabetic mouse livers. N=8-10, ^*^P<0.05 versus control mice, ^#^P<0.05 versus STZ-Ad-GFP mice. **(E-F)** FAM3C overexpression on the protein levels of metabolic genes in diabetic mouse livers. Con, control mice; STZ-Ad-GFP, STZ-treated mice transduced with Ad-GFP; STZ-Ad-FAM3C, STZ-treated mice transduced with Ad-FAM3C. N=6-8, ^#^P<0.05 versus STZ-Ad-GFP mice.

To directly evaluate the effects of FAM3C-HSF1-CaM pathway on hepatic gluconeogenesis and hyperglycemia of type 1 diabetic mice, FAM3C or HSF1 was overexpressed in STZ-treated mouse livers via tail vein injection of Ad-FAM3C or HSF1 plasmid. At 3 and 7 days post Ad-FAM3C injection, the fasting blood glucose levels were significantly reduced when compared with mice treated with Ad-GFP (Figure 2A). Particularly, injection of Ad-FAM3C almost reduced the fasting blood glucose levels of diabetic mice to that of normal mice post 7 days (Figure 2A). The serum insulin levels between Ad-GFP- and Ad-FAM3C-treated mice were not different (Figure 2B). The mRNA levels of insulin genes in Ad-GFP- and Ad-FAM3C-treated mouse pancreas were also not different (Figure 2C). Injection of Ad-FAM3C increased the mRNA levels of HSF1 and CALM1, while reduced that of PEPCK and G6Pase in diabetic mouse livers (Figure 2D). Injection of Ad-FAM3C increased both FAM3C protein isoforms in diabetic mouse livers (Figure 2E-2F). FAM3C overexpression increased the protein levels of HSF1, CaM and phosphorylated Akt (pAkt) with the reduced PEPCK and G6Pase protein levels in type 1 diabetic mouse livers (Figure 2E-2F).

**Figure 3:**
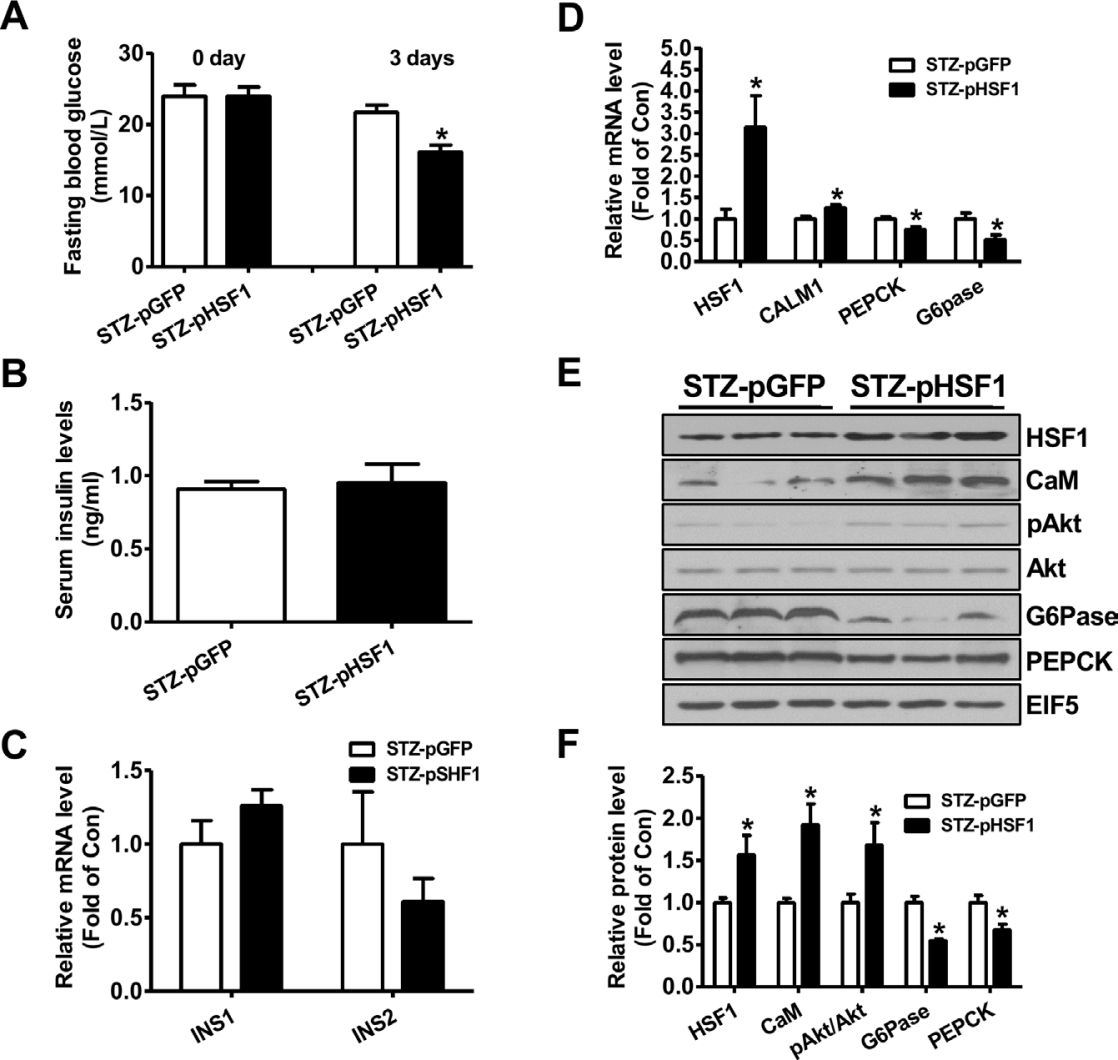
Plasmid overexpression of HSF1 in the livers improved hyperglycemia of type 1 diabetic mice Diabetic mice were transfected with plasmid as described in the experimental procedure. **(A)** Fasting blood glucose levels of diabetic mice before and after plasmid transfection. **(B)** Serum insulin levels after plasmid transfection. **(C)** Insulin mRNA expression levels in pancreas of mice after plasmid transfection. **(D)** Injection of HSF1 plasmid on the mRNA levels of metabolic genes in diabetic mouse livers. N=8, ^*^P<0.05 versus mice STZ-pGFP mice. **(E-F)** HSF1 overexpression on the protein levels of metabolic genes in diabetic mouse livers. STZ-pGFP, STZ-treated mice transfected with GFP plasmid; STZ-pHSF1, STZ-treated mice transfected with HSF1 plasmid. N=6-8, versus STZ-pGFP group of mice.

At 3 days post HSF1 plasmid injection, the fasting blood glucose levels were decreased when compared with diabetic mice treated with pGFP plasmid (Figure 3A). Injection of HSF1 plasmid had little effect on serum insulin levels and insulin gene expression in pancreas (Figure 3B-3C). Injection of HSF1 plasmid increased the mRNA levels of HSF1 and CALM1, while reduced that of PEPCK and G6Pase in type 1 diabetic mouse livers (Figure 3D). HSF1 overexpression increased the protein levels of CaM and pAkt but decreased that of PEPCK and G6Pase in diabetic mouse livers (Figure 3E-3F). Overall, the changes in hepatic Akt phosphorylation and gluconeogenic gene expression were consistent with the amelioration of hyperglycemia after FAM3C or HSF1 overexpression.

### FAM3C secretion is necessary for its effects on Akt activation and gluconeogenesis repression in cultured hepatocytes

**Figure 4:**
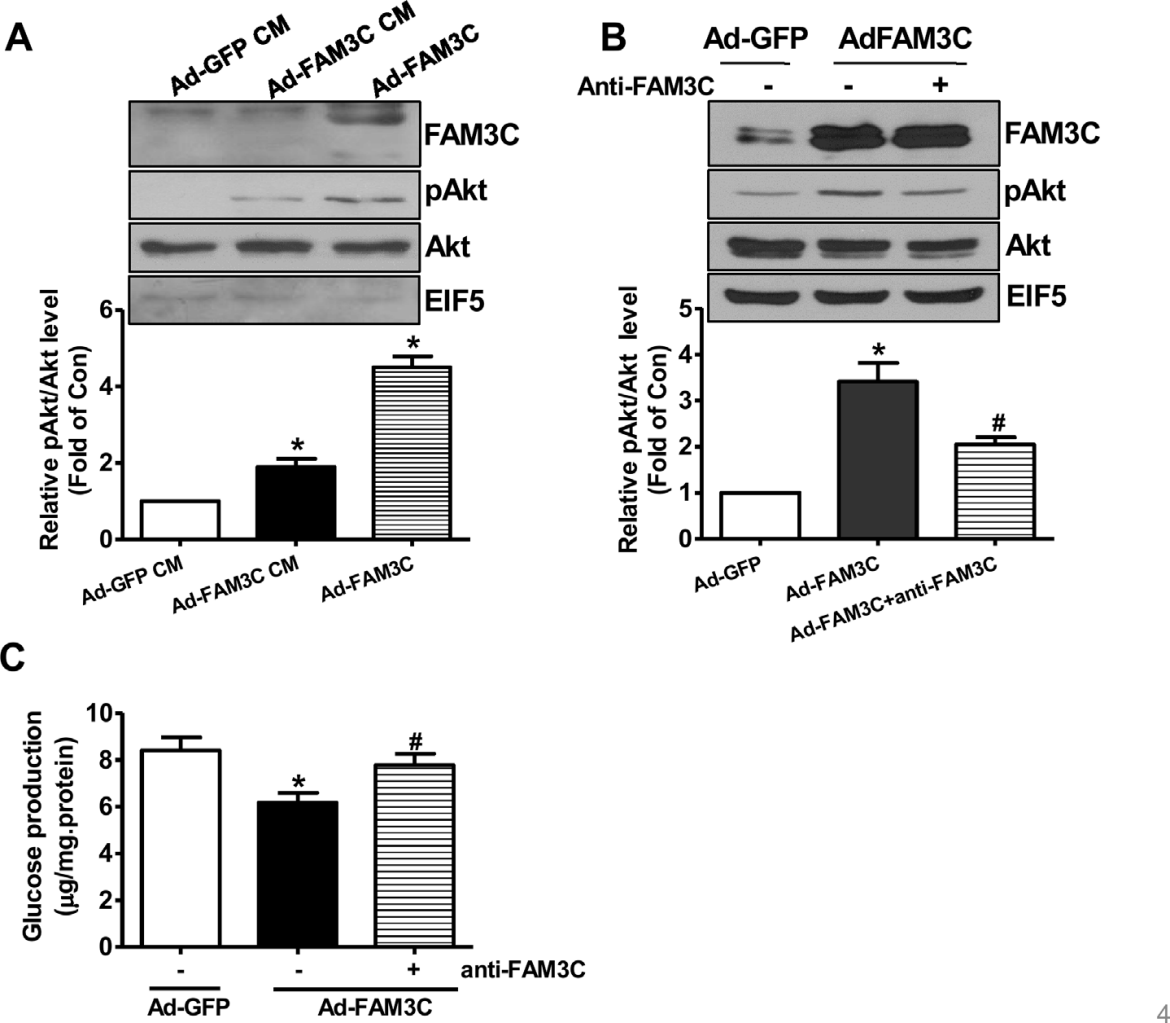
Secretion is important for FAM3C-induced Akt activation and gluconeogenesis repression in HepG2 cells **(A)** Conditioned medium of Ad-FAM3C-infected cells induced Akt phosphorylation in HepG2 cells. HepG2 cells were treated with conditioned medium of Ad-GFP- or Ad-FAM3C-infected cells for 24 hours before Akt phosphorylation was assayed. Ad-FAM3C-infected cells was used as a positive control. N=3, ^*^P<0.05 versus cells treated with conditioned medium of Ad-GFP-infected cells. **(B)** Treatment with anti-FAM3C antibodies inhibited Akt activation induced by FAM3C overexpression in HepG2 cells. **(C)** Treatment with anti-FAM3C antibodies reversed FAM3C-induced repression on gluconeogenesis in HepG2 cells. CM, conditioned medium. N=4-5, ^*^P<0.05 versus Ad-GFP-infected cells, ^#^P<0.05 versus Ad-FAM3C-infected cells.

**Figure 5:**
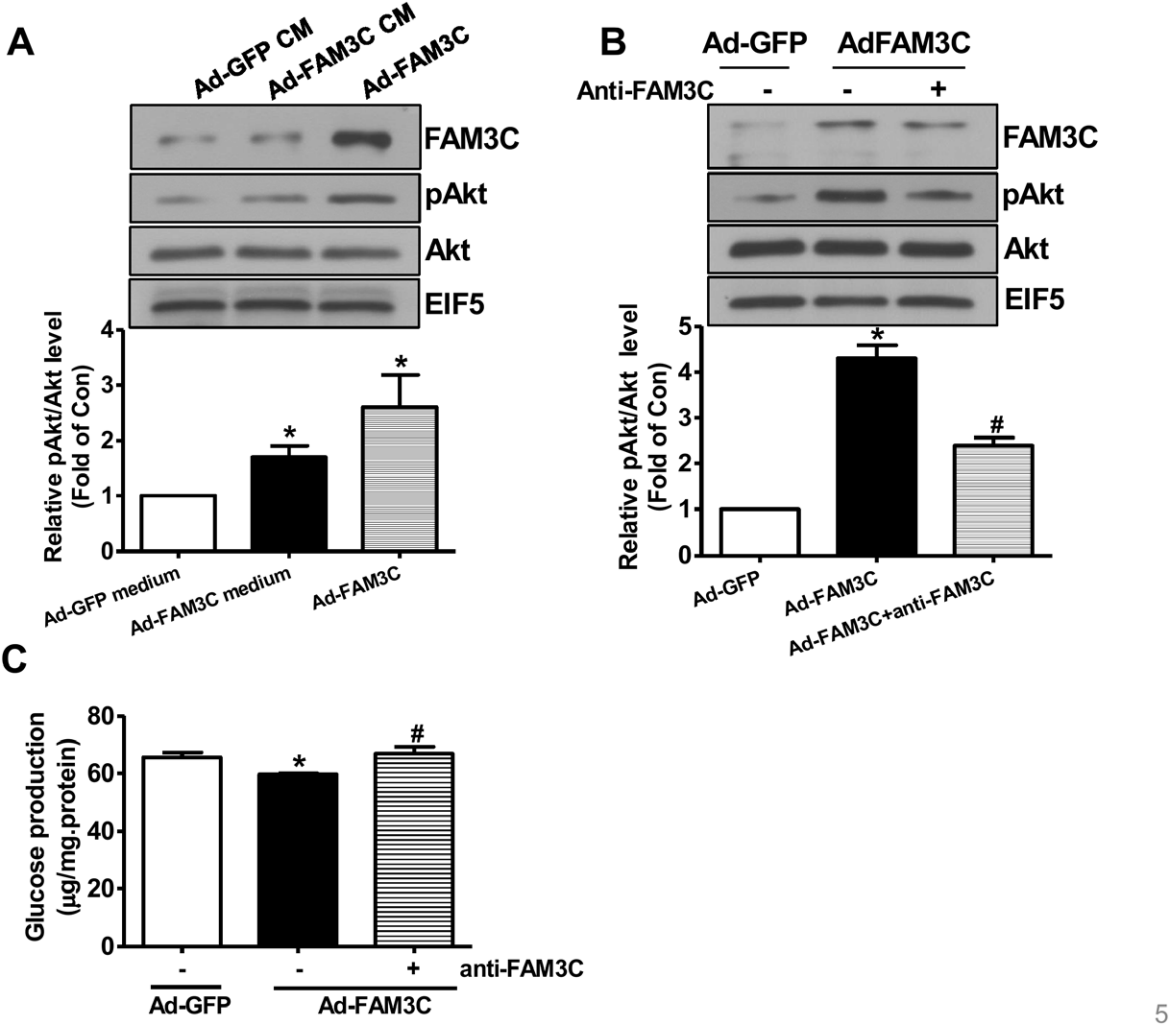
Secretion is imporant for FAM3C-induced Akt activation and gluconeogenesis repression in primary mouse hepatocytes **(A)** Conditioned medium of Ad-FAM3C-infected cells induced Akt phosphorylation in primary mouse hepatocytes. Mouse hepatocytes were treated with conditioned medium of Ad-GFP- or Ad-FAM3C-infected hepatocytes for 24 hours before Akt phosphorylation was assayed. Ad-FAM3C-infected hepatocytes was used as a positive control. N=3, ^*^P<0.05 versus cells treated with conditioned medium of Ad-GFP-infected cells. **(B)** Treatment with anti-FAM3C antibodies inhibited Akt activation induced by FAM3C overexpression in primary mouse hepatocytes. **(C)** Treatment with anti-FAM3C antibodies reversed FAM3C-induced repression on gluconeogenesis in primary mouse hepatocytes. CM, conditioned medium. N=4, ^*^P<0.05 versus Ad-GFP-infected cells, ^#^P<0.05 versus Ad-FAM3C-infected cells.

To determine whether or not secretion is necessary for FAM3C-induced Akt activation, HepG2 cells were treated with conditioned medium of Ad-GFP- or Ad-FAM3C-treated cells, respectively. As a result, conditioned medium of Ad-FAM3C-treated cells induced Akt activation in HepG2 cells (Figure 4A). In support, Akt activation induced by FAM3C overexpression was inhibited by treatment with anti-FAM3C antibodies in HepG2 cells (Figure 4B). Anti-FAM3C antibody treatment had little effect on FAM3C expression in the cells (Figure 4B). What's more, gluconeogenesis repression induced by FAM3C overexpression was also reversed by treatment with anti-FAM3C antibodies (Figure 4C). In support, conditioned medium of Ad-FAM3C-treated mouse hepatocytes also induced Akt phosphorylation in primary mouse hepatocytes (Figure 5A). Akt phosphorylation and gluconeogenesis suppression induced by FAM3C overexpression were also reversed by the treatment of anti-FAM3C antibodies in primary mouse hepatocytes (Figure 5B-5C).

### Recombinant FAM3C protein activated Akt pathway to repress gluconeogenesis in a HSF1- and CaM-dependent manner in hepatocytes

**Figure 6:**
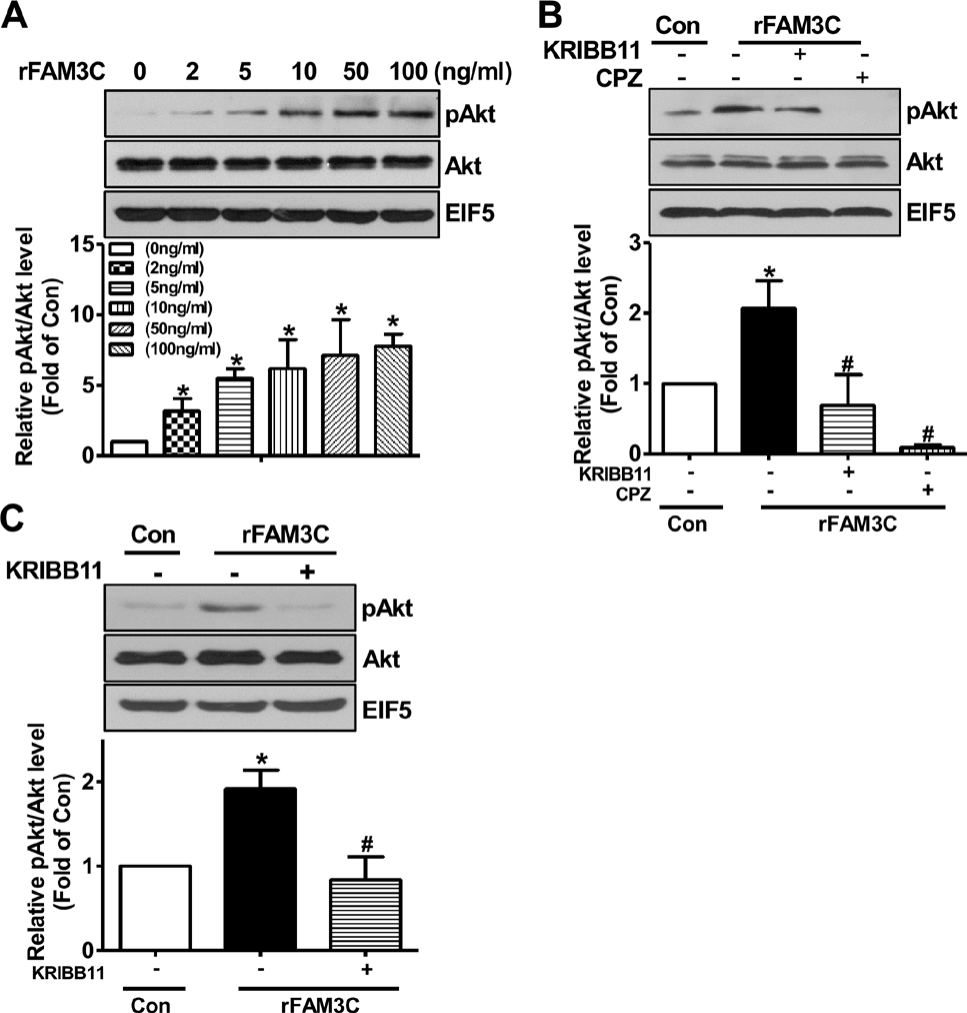
rFAM3C induced Akt phosphorylation in hepatocytes **(A)** rFAM3C dose-dependently induced Akt activation in HepG2 cells. Cells were treated with various concentration of rFAM3C for 24 hours. **(B)** rFAM3C induced Akt activation in HSF1- and CaM-dependent manner in HepG2 cells. Cells were treated with 5ng/ml rFAM3C for 24 hours. **(C)** rFAM3C induced Akt activation in HSF1-dependent manner in primary mouse hepatocytes. Hepatocytes were treated with 5ng/ml rFAM3C for 24 hours. The treatment of inhibitors was detailed in the experimental procedure. Con, control cells; rFAM3C, cells treated with rFAM3C. rFAM3C, recombinant human FAM3C protein. N=4, ^*^P<0.05 versus control cells, ^#^P<0.05 versus rFAM3C-treated cells without inhibitors.

To further confirm that secretion is necessary for FAM3C-induced activation of HSF1-CaM-Akt pathway, human HepG2 cells and mouse hepatocytes were treated with rFAM3C. As a result, rFAM3C dose-dependently induced Akt phosphorylation in HepG2 cells (Figure 6A). Importantly, Akt phosphorylation induced by rFAM3C was blocked by HSF1 inhibitor KRIBB11 and CaM inhibitor CPZ in HepG2 cells (Figure 6B). Moreover, rFAM3C similarly induced Akt phosphorylation in primary mouse hepatocytes (Figure 6C). Inhibition of HSF1 also abolished rFAM3C-induced Akt activation in primary mouse hepatocytes (Figure 6C).

**Figure 7:**
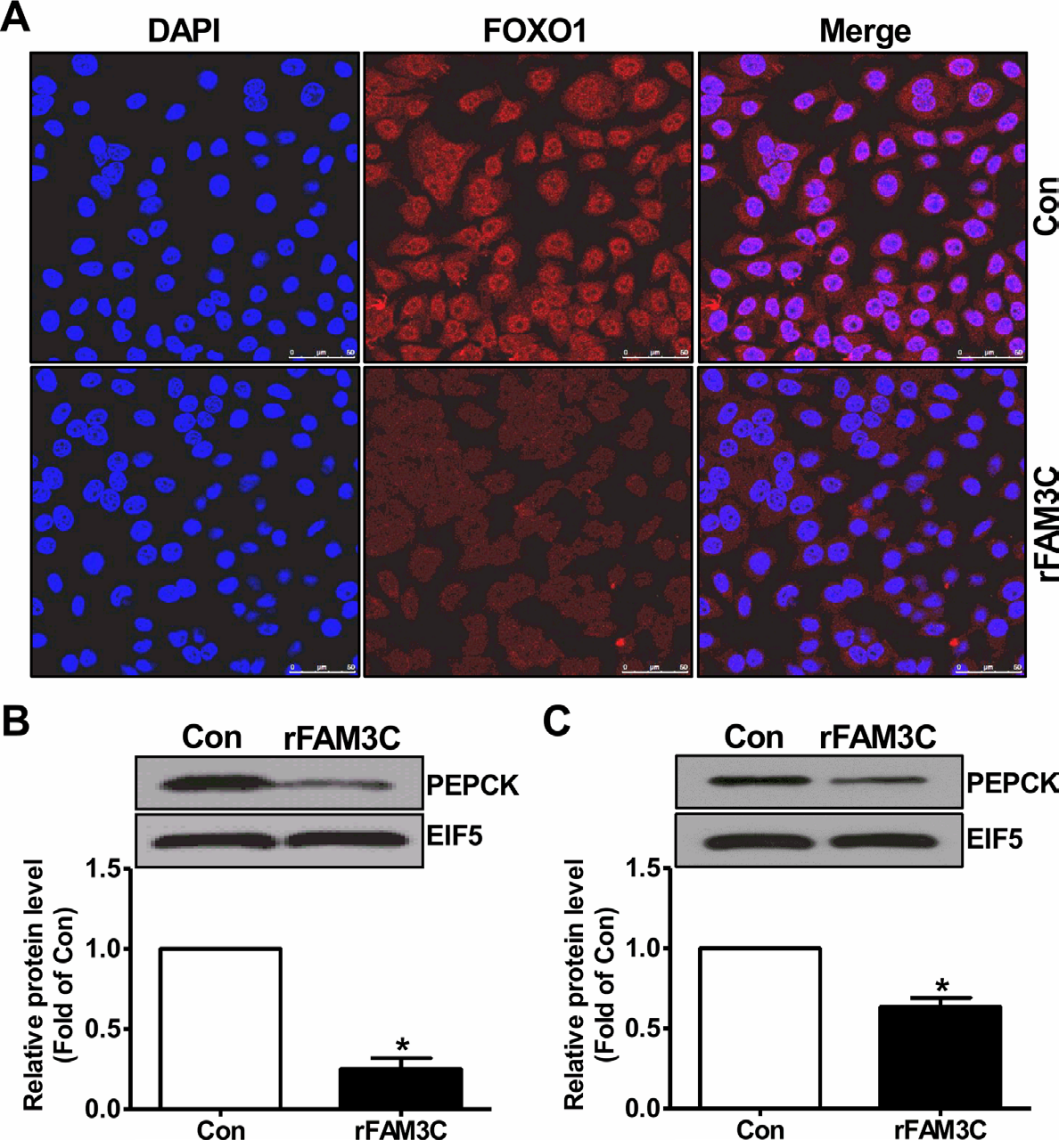
rFAM3C inactivated FOXO1 and repressed gluconeogenic gene expression in hepatocytes Cells were treated with 5ng/ml rFAM3C for 24 hours. **(A)** rFAM3C promoted the nuclear exclusion of FOXO1 in HepG2 cells. The images were the representatives of 3 experiments. **(B)** rFAM3C reduced PEPCK protein level in HepG2 cells. **(C)** rFAM3C reduced PEPCK protein level in primary mouse hepatocytes. Con, control cells; rFAM3C, cells treated with rFAM3C. N=4-5, ^*^P<0.05 versus control cells.

**Figure 8:**
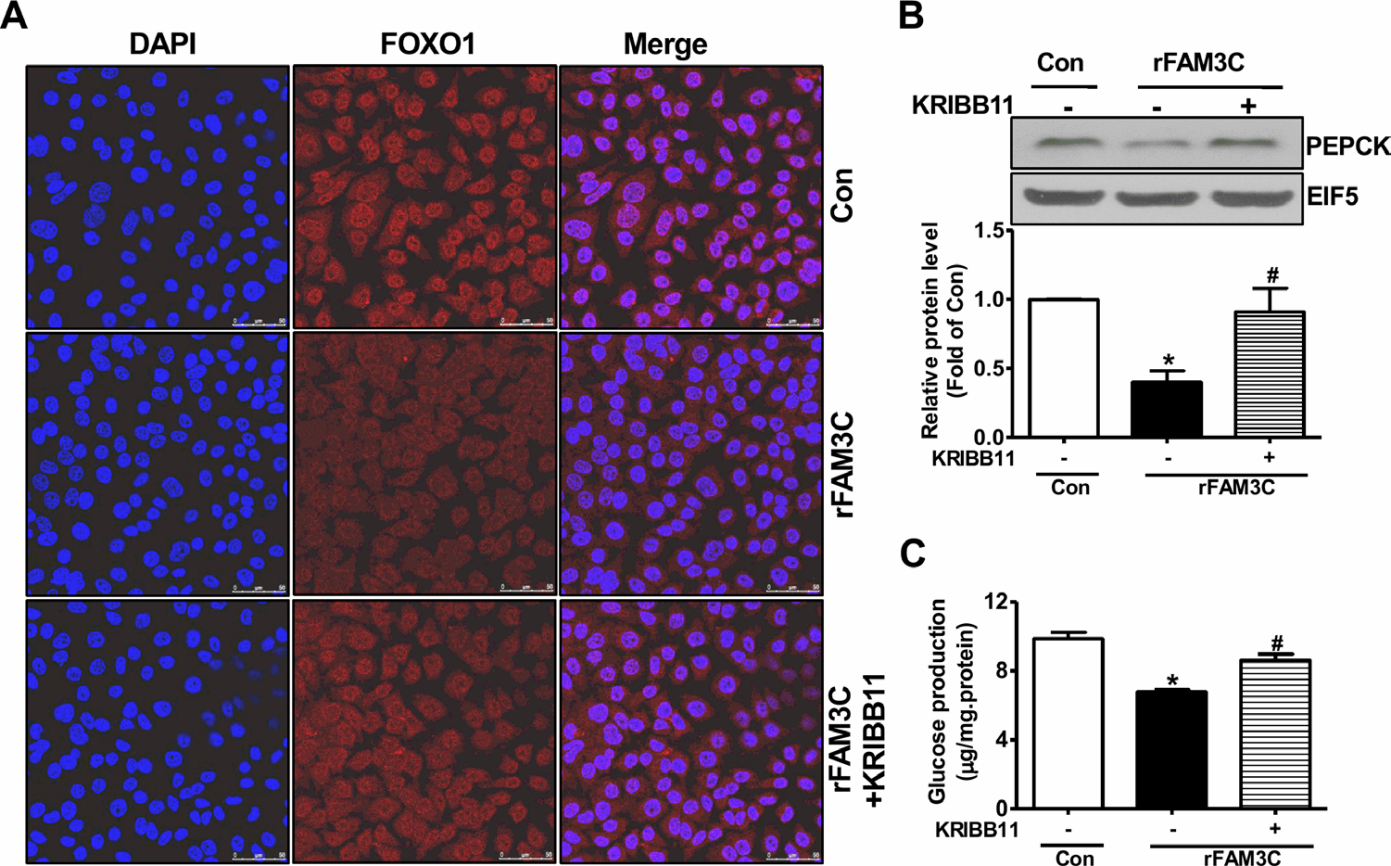
rFAM3C inactivated FOXO1 and repressed gluconeogenic gene expression in HSF1-dependent manner Cells were treated with 5ng/ml rFAM3C for 24 hours in the absence or presence of KRIBB11. **(A)** rFAM3C promoted the nuclear exclusion of FOXO1 in HSF1-dependent manner in HepG2 cells. The images were the representatives of 3 experiments. **(B)** rFAM3C repressed PEPCK protein expression in HSF1-dependent manner in HepG2 cells. **(C)** rFAM3C repressed gluconeogenesis in HSF1-dependent manner in HepG2 cells. Con, control cells; rFAM3C, cells treated with rFAM3C. N=4-5, ^*^P<0.05 versus control cells, ^#^P<0.05 versus rFAM3C-treated cells without inhibitor.

Physiologically, activation of Akt suppresses gluconeogenic gene expression and gluconeogenesis by promoting the nuclear exclusion of FOXO1, a key transcriptor controlling the transcription of crucial gluconeogenic genes such as PEPCK [4]. Consistent with the Akt activation, rFAM3C treatment promoted the nuclear exclusion of FOXO1 in HepG2 cells (Figure 7A). In support of FOXO1 inactivation, rFAM3C significantly reduced the PEPCK protein expression in HepG2 cells (Figure 7B). Moreover, rFAM3C also repressed PEPCK expression in primary mouse hepatocytes (Figure 7C). rFAM3C-triggered FOXO1 nuclear exclusion was further shown to be blocked by the inhibition of HSF1 using KRIBB11 in HepG2 cells (Figure 8A). Moreover, rFAM3C-induced repression on PEPCK expression was also reversed by inhibiting HSF1 (Figure 8B). In support of repression of FOXO1 activity and PEPCK expression, rFAM3C significantly repressed glucose production (Figure 8C). Importantly, rFAM3C-induced repression on gluconeogenesis was also reversed by inhibiting HSF1 in HepG2 cells (Figure 8C). Overall, rFAM3C induced Akt activation and repressed gluconeogenesis in hepatocytes via HSF1-CaM pathway as FAM3C overexpression [19].

## DISCUSSION

**Figure 9:**
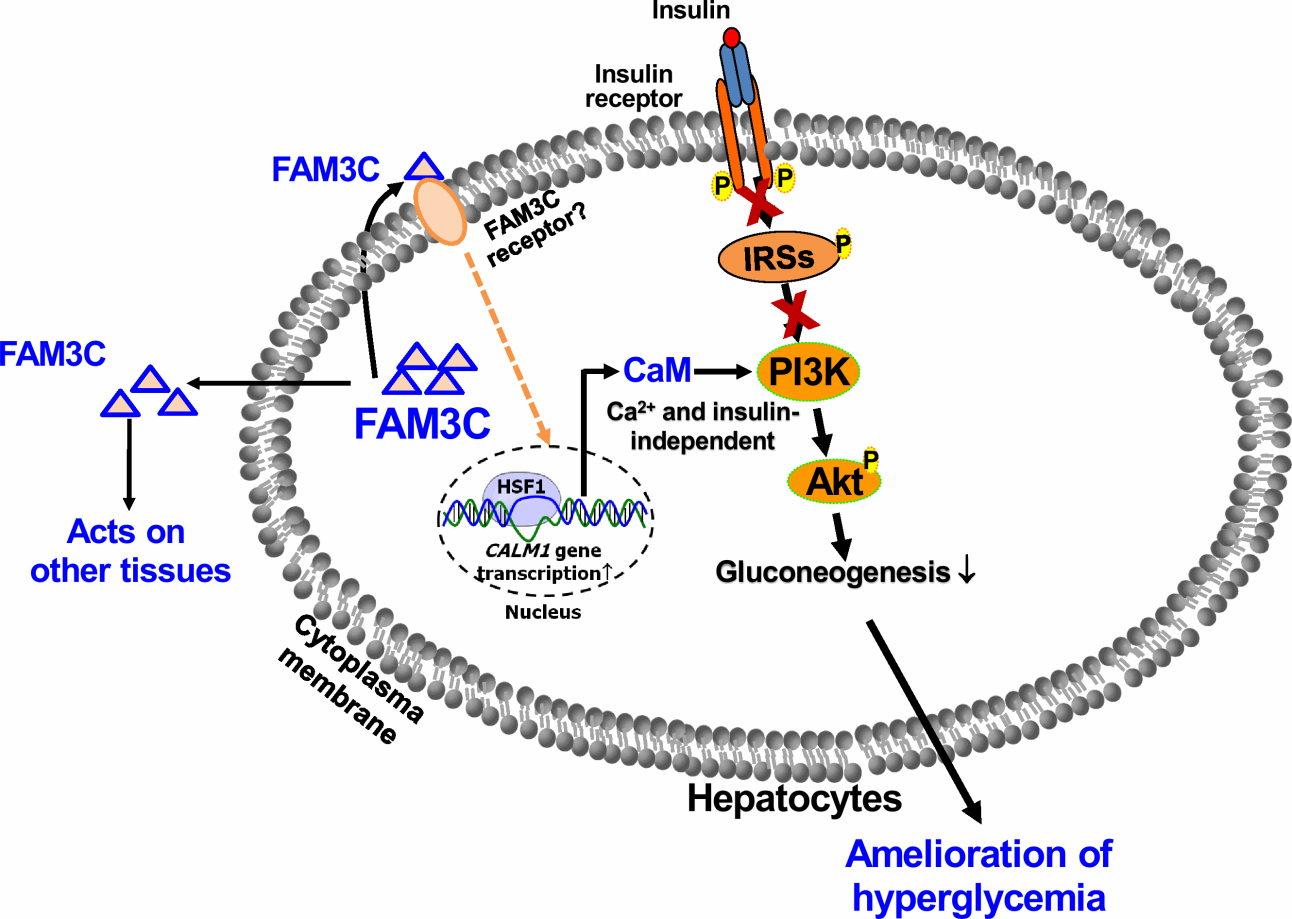
FAM3C functions as a hepatokine to suppress hepatic gluconeogenesis independent of insulin FAM3C is a new hepatokine that activates HSF1-CaM-PI3K pathway to induce Akt phosphorylation independent of insulin and calcium. Liver-secreted FAM3C may also act on other tissues beyond liver to exert its metabolic effects. Under severe insulin resistant and/or insulin deficient status, activating hepatic FAM3C-HSF1-Akt pathway represents a novel strategy for the treatment of diabetes.

In the current study, we provided new evidences that FAM3C activated HSF1-CaM-Akt pathway to repress hepatic gluconeogenic gene expression and attenuate hyperglycemia of type 1 diabetic mice. These findings and the previous findings together revealed that FAM3C suppresses hepatic gluconeogenesis independent of insulin *in vitro* and *in vivo*. What's more, we provided additional evidences that secretion of FAM3C protein is important for its effects of inducing Akt phosphorylation and repressing gluconeogenesis in cultured human and mouse hepatocytes. Clearly, FAM3C is a new hepatokine. Our previous and current findings revealed hepatic FAM3C expression will regulate glucose metabolism by impacting on liver and other tissues as both an autocrine and an endocrine factor (Figure 9). So far, the FAM3C receptor still remains unknown. To identify the FAM3C receptor and determine its tissue distribution will be crucial for understanding FAM3C signaling transduction and its overall effects in glucose metabolism. This also suggested that circulating FAM3C could be a new biomarker for diagnosis and classification of diabetes, particularly in the era of precision medicine. Beyond diabetes, dysregulated glucose and lipid metabolism are also highly associated with various cancers [22]. FAM3C expression is upregulated in several cancers, and circulating FAM3C level could also be a biomarker for autophagy and some cancers [23–25]. Anti-diabetic drug metformin exerts beneficial effects in various cancers in animals and humans [26]. Under conditions with dysregulated glucose and lipid metabolism, serum FAM3C protein level might be a novel biomarker for predicting the risk of diabetes or cancer. So far, there is no effective and sensitive method for detecting secreted FAM3C protein in the circulation of human and animals, and in cell culture medium. To develop high-sensitive FAM3C detection kit is necessary for establishing FAM3C as a new biomarker for both diabetes and cancer. PANDER (FAM3B) is processed by cleaving the signal peptide and then secreted from pancreatic alpha and beta cells, and secreted PANDER induces hepatic insulin resistance [14, 27, 28]. An elevation in circulating PANDER level is associated with insulin resistance and hyperglycemia in a Chinese population [29]. However, although liver-produced PANDER also plays important roles in regulating glucose and lipid metabolism, only one PANDER protein isoform (full length form, 27kD) can be detected in mouse livers and cultured hepatocytes with or without PANDER overexpression [9, 10, 13, 30], suggesting that PANDER may not be processed and released from hepatocytes.

Recently, it had been realized that cellular stress response plays important roles in the pathogenesis of type 2 diabetes [31, 32]. When cells are exposed to stressors such as heat, radiation and oxidative stress, the expression of heat shock proteins (HSP) will be induced to protect cells against the damages triggered by these deleterious factors. These HSP are also called inducible HSP (iHSP) [31, 32]. The main functions of iHSP include assisting the folding of new or denatured proteins, preventing protein degradation and removing the nonfunctional proteins under stressed conditions [31–33]. As detailed in review [32], the expression levels of iHSP and their response to stressors are impaired in some important metabolic tissues including skeletal muscle and liver under diabetic condition. HSF1 is the key transcriptor controlling the transcription of iHSP, and decreased HSF1 expression in main metabolic tissues is highly associated with insulin resistance and diabetes [34, 35]. In support, HSP72 expression was reduced in muscle under obese and insulin resistant status, and muscle-specific HSP72 overexpression protects against obesity-induced insulin resistance and glucose intolerance. Moreover, chaperone co-inducer BGP-15 activates HSP72 to suppress hepatic glucose production, improve insulin resistance and hyperglycemia in obese diabetic mice [36]. Moreover, BGP-15 increases HSF1 binding activity and enhances the heat shock response through increased chromatin accessibility [37]. In the current and previous studies [19], we provided strong evidences that FAM3C function as a new hepatokine. FAM3C activates HSF1 to induce CALM1 expression and elevate CaM protein level, which finally promotes Akt phosphorylation and suppresses gluconeogenesis independent of insulin in hepatocytes. In contrast, FAM3C has little effect on CALM2 and CALM3 transcription [19]. CALM1, CALM2 and CALM3 encode one identical CaM protein. New FAM3C-HSF1-CaM-Akt metabolic pathway shed light on the understanding of the metabolic roles of HSF1. Interestingly, activation of Akt has also been reported to activate HSF1 [31, 32], suggesting that a cross-regulation exists between HSF1 and Akt. Overall, these findings together strongly suggested that HSF1 represents a novel therapeutical target for treating diabetes and other metabolic diseases.

In conclusion, FAM3C is a new hepatokine that activates HSF1-CaM-Akt pathway to ameliorate hyperglycemia of type 1 diabetic mice by suppressing hepatic gluconeogenesis in an insulin-independent manner (Figure 9). FAM3C-HSF1 axis is the potential target for the treatment of type 1 and type 2 diabetes.

## MATERIALS AND METHODS

### Construction of type 1 diabetic mouse models

8-10 week old male C57BL/6 mice were used in the current study. Type 1 diabetes (T1D) was induced by intraperitoneal injection of streptozotocin (STZ) at the dose of 50mg/kg.bodyweight for 7 days. Mice with fasting blood glucose levels greater than 16.4mmol/L were considered diabetic one month later. The control mice were treated with saline. Animal experiments were conducted in accordance with the Animal Management Rules of the Ministry of Health of the People's Republic of China and the guide for the Care and Use of the Laboratory Animals of the Peking University.

### Adenoviral overexpression of FAM3C in T1D mouse livers

To overexpress FAM3C in T1D mouse livers, 1.0×10^9^ pfu of Ad-FAM3C or Ad-GFP were injected into mice via tail vein. Fasting blood glucose levels were monitored at the 4^th^ and 7^th^ day post viral injection. On the 8^th^ day, the mice were sacrificed on fed state for the experimental assays. The serum was collected for analyzing insulin levels. Liver and pancreas were taken for biochemical analyses.

### Hydrodynamics-based plasmid overexpression of HSF1 in T1D mouse livers

Hydrodynamics-based transfection in animals by tail-vein administration of naked plasmid DNA was detailed previously [16, 19]. In brief, STZ-treated diabetic mice were randomly divided into two groups based on fasting blood glucose levels. 50μg endotoxin-free pGFP or pHSF1 was dissolved in sterile saline with the volume 10% of the bodyweight at room temperature, and then injected into tail vein in 7 seconds. Fasting blood glucose was monitored at 72 hours post plasmid injection. 24 hour later, mice were sacrificed for assays as above. The serum was collected for insulin detection.

### Conditioned medium and anti-FAM3C antibody treatment experiments

HepG2 cells or mouse primary hepatocytes were infected with Ad-GFP or Ad-FAM3C for 24 hours, and then the cell culture medium was collected. The medium was centrifuged at 13,000g at 4°C for 20 minutes, and the supernatant was collected as conditioned medium. Conditioned medium was added into cultured HepG2 cells or mouse hepatocytes to 1/3 volume of the culture medium, and the cells were incubated for 24 hours before being lysed for Akt phosphorylation analyses. For anti-FAM3C antibody treatment experiment, HepG2 cells were infected with Ad-FAM3C for 6 hours, and then incubated with anti-FAM3C antibodies (Abcam, ab72182) at the concentration of 2.5μg/ml for 24 hours before pAkt or glucose production analyses. The procedure for gluconeogenesis assay in cultured hepatocytes was detailed in previous studies [16, 19].

### Treatment with recombinant human FAM3C protein

Recombinant human FAM3C protein (rFAM3C) was purchased from Novoprotein (Cat#:C343, secretory form, 22kD). Human HepG2 cells or primary mouse hepatocytes were treated with indicated concentration of rFAM3C for 24 hours before experimental analyses. For inhibiting HSF1, cells were treated with rFAM3C in the absence or presence of KRIBB11 (3μM) for 24 hours. For inhibiting CaM, cells treated with rFAM3C for 24 hours were incubated with CPZ (100μM) for 1 hour before pAkt analyses.

### Primary mouse hepatocyte culture

Livers were perfused *in situ* with 50ml of KRB buffer solution, followed by 30ml of liver digestion medium containing collagenase. The liver was excised, minced, and strained through a steel mesh. The dispersed hepatocytes were collected by centrifugation at 50g for 3 min at 4°C for three times. Isolated hepatocytes were washed and plated in collagen-coated plates at 37°C, 5% CO_2_ for 6-8 h to allow for attachment, followed by the removal of unattached cells and treatment. Hepatocytes were treated with recombinant FAM3C protein for 24 hours before analyses.

### RNA extraction and real time-PCR assays

Total RNA from HepG2 cells or tissues was prepared using TRIzol (Invitrogen) in accordance with the manufacturer's recommendations. 2μg total RNA was used in each cDNA synthesis reaction using cDNA Synthesis Kit (Thermo Scientific) with an oligo (dT) primer. Quantitative real-time PCR was performed using SYBR Green PCR Master Mix. The amplification was performed at 94°C for 2 minutes and subjected to 40 cycles of 94°C for 30 seconds, 59°C for 30 seconds, and 72°C for 30 seconds, followed by a final extension at 72°C for 6 minutes. The relative levels of the target gene mRNA transcripts to the β-actin were calculated by 2^−ΔΔCt^ methodology as detailed previously [15, 16, 21]. All PCR primers were listed in previous study [19].

### Western blotting

Cells or tissues were lysed in RIPA lysis buffer containing proteinase inhibitors, followed by centrifugation for 10 minutes at 4°C 12,000 rpm to collect supernatants. Protein concentration was assessed with BCA protein assay kit. 40-100μg protein were separated by SDS-PAGE, followed by transferring to nitrocellulose membranes and blocking with 5% fat-free milk in TBS for 1 hours. The membranes were incubated with primary antibodies overnight at 4°C, followed by washing with TBS and incubating with a horseradish peroxidase-conjugated secondary antibody for 2 hours. Proteins were visualized with enhanced chemiluminescence technique. Akt phosphorylation was referred to phosphorylation at Ser473.

### Confocal analyses of FOXO1 nuclear exclusion

Cells treated with recombinant FAM3C were permeabilized with 0.2% Triton X-100/0.5% BSA, followed by washing with PBS. The coverslips were blocked in 1% BSA for 30 minutes at 37°C. The coverslips were incubated with anti-FOXO1 antibodies at 4°C overnight, and then washed with PBS, followed by detecting with goat anti-rabbit Alexa Fluor 594. After nuclear staining with DAPI, coverslips were mounted on glass slides using 50% glycerol in PBS. Mounted coverslips were imaged and cells were visualized by fluorescence microscopy using Confocal Laser Scanning Microscope.

### Statistical analyses

The results are presented as the mean ± SEM. Statistical significance of differences between groups was analyzed by *t*-test. P values <0.05 were considered as statistically significant.
